# Sputnik‐V vaccine‐induced panniculitis as a local reactions

**DOI:** 10.1002/ccr3.5923

**Published:** 2022-06-02

**Authors:** Zahra Sahraei, Bahareh Abtahi‐Naeini, Ali Saffaei

**Affiliations:** ^1^ Department of Clinical Pharmacy School of Pharmacy Shahid Beheshti University of Medical Sciences Tehran Iran; ^2^ Skull Base Research Center Shahid Beheshti University of Medical Sciences Loghman Hakim Hospital Tehran Iran; ^3^ Pediatric Dermatology Division of Department of Pediatrics Imam Hossein Children's Hospital, Isfahan University of Medical Sciences Isfahan Iran; ^4^ 48455 Skin Diseases and Leishmaniasis Research Center Isfahan University of Medical Sciences Isfahan Iran

**Keywords:** adverse drug reaction, COVID‐19, panniculitis, Sputnik‐V, vaccine

## Abstract

Here, a case of Sputnik‐V vaccine‐induced panniculitis was reported. The patient developed erythema, induration, and local tenderness at the injection site after 13 days of the injection. Ultra‐sonography imaging showed inflammation in subcutaneous layers including fat tissue compatible with panniculitis. She received ibuprofen and warm compress, and all symptoms resolved.

## Graphical abstract

COVID‐19 vaccines have a variety of side effects, from topical to systemic. Some local side effects of these vaccines are known, but some are unknown and occur rarely. Panniculitis as a local adverse event should be kept in the mind of physicians.

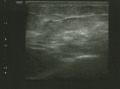

## 1 INTRODUCTION

Up to now, several vaccines against SARS‐CoV‐2 have been developed. There are different platforms for COVID‐19 vaccines such as virus vector vaccines, protein subunit vaccines, and monoclonal antibodies therapy.^1^ They have their discrete benefits and hindrances. Pfizer‐BioNTech and Moderna are messenger RNA (mRNA) vaccines, which introduced as the first COVID‐19 vaccines. Other vaccines such as Johnson & Johnson, Astra‐Zeneca, and Sputnik‐V use human and primate adenovirus vectors. Sinovac and Sinopharm are inactivated vaccines. As of January 2021, one hundred seventy‐three vaccines are in preclinical development and 64 in clinical trials.^2^ The safety data were published for 11 vaccines as interim reports or clinical trial reports. The frequently reported local adverse events were pain at the injection site, swelling, and redness. The systemic adverse events included fever, fatigue, myalgia, and headache. Some studies also reported decreased hemoglobin, increased bilirubin, and altered liver enzymes.^3^ The most common skin reactions were redness/erythema, itchiness, urticarial rash, morbilliform eruptions, pityriasis rosea, swelling, and burning.^4^ It is very important to establish the safety of the COVID‐19 vaccines when emergency approval is being granted to these vaccines without the completion of all phases of clinical trials. Since vaccines are still being tested in clinical trials, it is necessary to evaluate the unusual adverse effects of vaccines. Here, we reported an unusual adverse effect of the Sputnik‐V vaccine.^5^

## 2 CLINICAL HISTORY

A 40‐year‐old woman who was a healthcare provider received the first dose of the Sputnik‐V vaccine on March 1, 2021, via intramuscular injection on the left deltoid muscle. She was a healthy woman without any notable past medical history. She was not taking any medicines. She developed mild constitutional symptoms and pain on site of injection about 12 h after vaccination. All symptoms were tolerable and revealed during the next 24 h except for local pain. After 13 days of the first injection, she complained of severe pain and induration at the site of injection, which limits any movement of the arm. The clinical examination showed minimal erythema and obvious induration associated with local tenderness of the left hand beyond the injection site of the vaccine. This affected area was about 5 × 10 cm. For rolling out of any collection and abbesses formation, soft tissue ultra‐sonography was requested.

**FIGURE 1:**
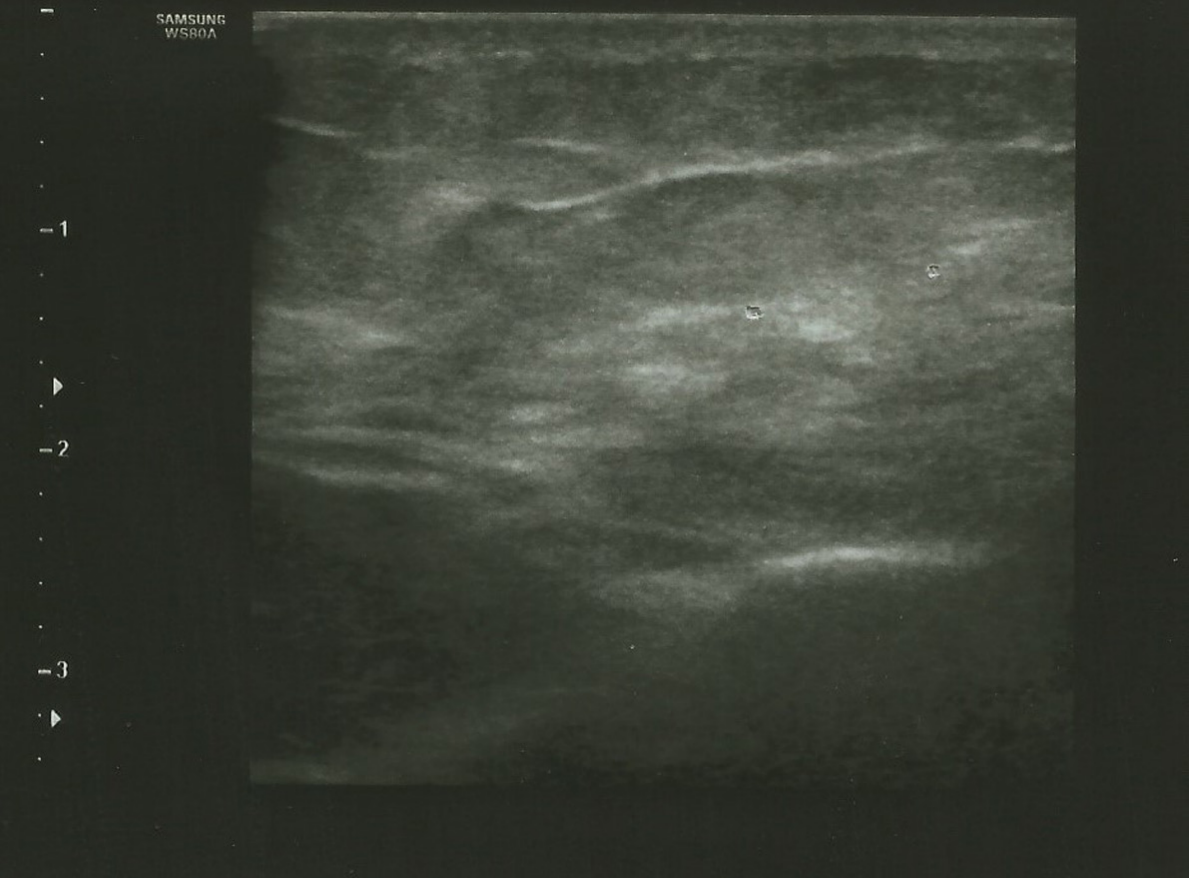
The ultra‐sonography imaging of Sputnik‐V vaccine‐induced panniculitis

Ultra‐sonography imaging showed inflammation in subcutaneous layers including fat tissue compatible with panniculitis (Figure 1).

The patient did not allow us to take a biopsy, according to the clinical and para‐clinical findings the diagnosis of Sputnik‐V vaccine‐induced panniculitis as a severe and delayed form of COVID‐19 arm to the Sputnik‐V vaccine was made for the patients. She received treatment for her symptoms with a warm compress and ibuprofen 400 mg every 4 h for three days. The patient was observed and all symptoms resolved gradually. After 3 months of follow‐up, there is no clinical and sonographic feature of any inflammation.

## 3 DISCUSSION

This case was the first report of delayed large local reactions to the Sputnik‐V vaccine. A similar report was published by Blumenthal et al^6^ They reported delayed large local reactions to mRNA‐1273 vaccine in 12 patients. The diagnosis of this adverse effect should be made cautiously. Cellulitis is an important differential diagnosis in such cases. However, we excluded cellulitis in our case because of the fat layers involved in ultra‐sonography imaging. This situation is less common in cellulitis. The time between injection and the development of adverse effects is also too long to consider cellulitis.

Although it cannot be ignored that in most patients with COVID‐19 arm may represent minimal self‐limited local reaction, severe and unusual local reactions also can be occurred.

The patient refused the skin biopsy due to a phobia of biopsy‐taking procure. In addition, she was anxious about this adverse effect and no more intervention was possible. Hence, a definite diagnosis was not possible. Other causes such as inappropriate injection techniques should be kept in the mind in such cases.

This case emphasizes the importance of these adverse effects and correct diagnosis of such cases can lead to avoiding unnecessary antibiotic prescriptions. Based on the above interpretations, we conclude that the local effects of COVID‐19 vaccines should be carefully evaluated because they can cause a wide range of side effects. These side effects can also be accelerated or delayed. Accurate history, clinical examination, ultrasound examination, and biopsy can help make an accurate diagnosis. Antibiotics should also be avoided until the diagnosis is clear. We expect this letter boosts additional reporting on clinical and epidemiologic characteristics of local reactions to the Sputnik‐V as a currently emergency‐approved SARS‐COV‐2 vaccine.

## AUTHOR CONTRIBUTIONS

ZS was involved in the management of the case and introduced the idea. AS and BA conducted a literature review and were involved in the conceptualization. All authors were involved in the writing of the manuscript draft, and all authors approved the final version of the manuscript.

## CONFLICT OF INTEREST

None.

## CONSENT

The written consent form was obtained.

## Data Availability

Not applicable.
